# Trajectories of childhood internalizing and externalizing psychopathology and psychotic-like experiences in adolescence: A prospective population-based cohort study

**DOI:** 10.1017/S0954579415001108

**Published:** 2016-05

**Authors:** Kristin S. Lancefield, Alessandra Raudino, Johnny M. Downs, Kristin R. Laurens

**Affiliations:** a King's College London; b Sussex Partnership National Health Service Foundation Trust; c University of New South Wales; d Schizophrenia Research Institute

## Abstract

Adolescent internalizing and externalizing psychopathology is strongly associated with adult psychiatric morbidity, including psychotic disorders. This study examined whether internalizing or externalizing trajectories (continuity/discontinuity of symptoms) from middle childhood were associated with adolescent psychotic-like experiences (PLEs). Prospective data were collected from a community sample of 553 children (mean age = 10.4 years; 50% male) and their primary caregivers. Participants completed questionnaire reports of internalizing and externalizing psychopathology and PLEs at baseline, and again approximately 2 years later. Logistic regression was used to examine the association of adolescent PLEs with four trajectories of internalizing and externalizing psychopathology (persistent, incident, remitting, and none), controlling for a range of potential confounders and sampling bias. Significant associations were identified between adolescent PLEs and the incident internalizing (adjusted odds ratio [adj. *OR*] = 2.96; 95% confidence interval [CI] = 1.60–5.49) and externalizing psychopathology (adj. *OR* = 2.14; 95% CI = 1.11–4.14) trajectories, as well as the persistent internalizing (adj. *OR* = 1.90; 95% CI = 1.13–3.18) and externalizing (adj. *OR* = 1.81, 95% CI = 1.02–3.19) trajectories. Children with remitting psychopathology trajectories were no more likely to present later PLEs than those who never experienced psychopathology. While for many individuals symptoms and illness remit during development without intervention, this study provides important insights regarding potential targets and timing for delivery of early intervention and prevention programs.

The importance of establishing early intervention and prevention programs that reduce the number of young people who develop severe mental illnesses such as schizophrenia is recognized globally (Collins et al., 2011). As a result, there has been considerable interest in the early manifestations or antecedents of psychotic disorders, with the rationale that treating earlier or milder expressions of psychosis may be preventative (Poulton et al., 2014). Several targets suggested for intervention include subclinical psychotic-like experiences (PLEs; Fisher et al., 2013; Kelleher et al., 2012; Poulton et al., 2000), as well as childhood disorders in both the internalizing and externalizing spectra (Cannon et al., 2002; Kim-Cohen et al., 2003; Welham et al., 2009). Childhood PLEs predict a variety of adult nonpsychotic disorders, including affective and anxiety, drug use, personality, and organic mental disorders, but they are relatively more sensitive and specific predictors of later nonaffective psychoses/schizophrenia (Fisher et al., 2013; Werbeloff et al., 2012). Internalizing (anxiety and depression) and externalizing (opposition/conduct and attention) disorders of childhood are associated with a number of adult psychiatric illnesses (Copeland, Shanahan, Costello, & Angold, 2009; Kim-Cohen et al., 2003), including psychotic disorders. Preliminary evidence suggesting that internalizing and externalizing psychopathology may be causally related to PLEs offers the prospect of reducing psychotic symptoms using intervention studies that target internalizing and externalizing psychopathology in childhood and adolescence (Ames et al., 2014; Poulton et al., 2014).

To determine appropriate targets and timing for delivery of early intervention and prevention programs for psychosis, a better understanding is needed of the natural course of internalizing and externalizing disorders during childhood and their relation with PLE outcomes in adolescence. Epidemiological studies reveal significant differences in disorder prevalence across childhood and adolescent periods. For example, internalizing and externalizing psychopathology in early to middle childhood samples from Australia (13% aged 5–15 years; Sawyer et al., 2001), Asia (4%–14% aged 6–12 years; Matsuura et al., 1993), western mainland Europe (12% aged 8–11 years; Fombonne, 1994), and the United Kingdom (10% aged 5–15 years; Meltzer, Gatward, Goodman, & Ford, 2003) are less prevalent than in adolescent samples, where psychopathology is reported cross-culturally among one fifth of individuals aged 12–19 years. (Costello, Copeland, & Angold, 2011). While symptoms and illness remit during development in a majority of young people who experience psychiatric disorder (Hofstra, Van der Ende, & Verhulst, 2000), studies that focus on transitional periods reveal instability in prevalence rates (both increases and decreases) from childhood (<12 years) into adolescence (12–19 years; Costello et al., 2011; Rutter, Kim-Cohen, & Maughan, 2006).

Studies examining PLE outcomes that take into account such transitional periods are scarce, especially in terms of the associations between internalizing and externalizing psychopathology in the childhood-to-adolescence transition. A previous longitudinal investigation assessed the relationship between continuity of *overall* psychopathology from ages 5 and 14 years (incorporating internalizing, externalizing, and other symptoms) and delusion-like experiences in young adulthood at age 21 years (Scott, Martin, Welham, et al., 2009). Findings indicated that children whose psychopathology persisted between 5 and 14 years had a 4.5 times increased risk of delusional-like experiences in young adulthood relative to children who never experienced psychopathology, while children whose problems emerged between the ages of 5 and 14 years (incident trajectory) presented a 3.8 times increased risk. Children whose difficulties at age 5 resolved by age 14 years (remitting trajectory) were no more likely than children who never experienced psychopathology to present delusion-like experiences in young adulthood. Generally, investigations examining internalizing and externalizing psychopathology have favoured middle adolescence onward or incorporated wide age ranges that likely obscure age-period variant effects. These studies have shown consistent associations between PLEs and both internalizing and externalizing disorders during the adolescent years (Kelleher et al., 2012; Nishida et al., 2008; Scott, Martin, Bor, et al., 2009; Wigman et al., 2012), but have been limited by their cross-sectional designs. Longitudinal studies of internalizing and externalizing psychopathology suggest that persistence of these disorders is associated with poorer outcomes than transient psychopathology. For example, internalizing symptoms arising in childhood predict persisting psychopathology through adolescence to adulthood in birth cohorts (Goodwin, Fergusson, & Horwood, 2004). The MRC National Survey of Health and Development birth cohort demonstrated that 70% of adolescents who had internalizing disorders at both 13 and 15 years of age went on to have mental disorder at follow-up during adulthood, compared to 25% of those who were mentally healthy adolescents and 33% of those having internalizing disorders at only a single time point in adolescence (Colman, Wadsworth, Croudace, & Jones, 2007). Whether similar associations exist between longitudinal patterns (trajectories) of internalizing or externalizing psychopathology and PLEs in the transitional period from late childhood to adolescence is unclear. Findings within adolescent samples may diverge from the pattern associated with the childhood to adolescence transition. Higher levels of internalizing and psychopathology occur among adolescents who report persistent PLEs relative to those who report intermittent PLEs, and to a greater extent than children who do not report PLEs (Thapar et al., 2012). In cross-sectional investigations, increasing PLEs in adolescence are associated with increases in externalizing behaviors (Mackie et al., 2013) and internalizing psychopathology (Wigman et al., 2012), but this is not always reflected in longitudinal data (Wigman et al., 2011). By contrast, in childhood, externalizing and internalizing psychopathology at age 5 years predicts PLEs at age 12 years (Polanczyk et al., 2010).

As far as we know, no studies have used a longitudinal design to examine the diverse internalizing and externalizing psychopathology trajectories that characterize the transitional period from late childhood to adolescence in relation to PLEs outcomes. This study reports data from a prospective community sample of children using both parent- and self-rated measures as they transition into adolescence. The study builds on evidence from prior work that shows that PLEs and internalizing and externalizing symptomology are dissociable constructs in childhood (age 9–11 years; Laurens, Hobbs, Sunferland, Green, & Mould, 2012). The aim was to test several hypotheses regarding the relationship between the stability/instability of internalizing and externalizing psychopathology over the transition from late childhood into adolescence and PLEs in adolescence. Relative to children who remained free of internalizing or externalizing psychopathology throughout the follow-up period, we hypothesized the following:

a. children with persisting internalizing or externalizing psychopathology would show an increased likelihood of reporting PLEs in adolescence;
b. children with internalizing or externalizing psychopathology arising de novo (incident) during the follow-up period would also show increased likelihood of reporting PLEs in adolescence, though not of the magnitude anticipated for individuals experiencing persisting psychopathology; and
c. children who reported internalizing or externalizing psychopathology at initial (baseline) assessment that remitted during the follow-up period would report equivalent prevalence of PLEs in adolescence.

## Methods

### Sample and recruitment

Longitudinal data were collected at two assessments. The first assessment, providing baseline data, was completed in 73 primary schools from the Greater London area between 2005 and 2010, as described previously (Laurens et al., 2007, 2012; Laurens, West, Murray, & Hodgins, 2008). Briefly, 8,099 children aged 9–11 years (mean age = 10.4 years, *SD* = 0.8 years; 50% male; comprising 95% of eligible children enrolled at participating primary schools) completed questionnaires independently in class, with corresponding questionnaires completed by the child's primary caregiver at home and returned via reply-paid mail (*n* = 1,504; 19%). Among these, 850 caregivers provided consent and identifying information for recontact purposes. The second assessment, providing follow-up data, was conducted between 2007 and 2012, with the subsample of 670 caregiver–child dyads whose contact information remained valid at the time of follow-up. Follow-up participants were invited to complete questionnaires individually at home or at the research institute, and received a £5 book voucher for participation. One hundred nine (16%) families refused participation at follow-up, with questionnaire responses supplied by 561 families, among whom 553 children and caregivers (83%) provided full data and constitute the longitudinal sample for analysis.

Ethical approval for the respective research phases (baseline and follow-up) were provided by the Joint South London and Maudsley and the Institute of Psychiatry National Health Service Research Ethics Committee and the King's College London Research Ethics Committee.

### Measures

#### Internalizing and externalizing trajectories

Internalizing and externalizing psychopathology were assessed via the self- and parent-report versions of the 25-item Strengths and Difficulties Questionnaire (SDQ; Goodman, 1997, 2001). The SDQ comprises four psychopathology scales assessing emotional symptoms, peer relationship problems, conduct problems, and hyperactivity–inattention, and a fifth subscale (prosocial behavior) assessing personal strengths. Sound psychometric properties for parent-report SDQ (ages 4–16 years) and self-report SDQ (ages 11–16 years) are established in community and clinical samples (Goodman, Lamping, & Ploubidis, 2010; Goodman, 2001). Self-report versions have been utilized with children as young as 8 years (Di Riso et al., 2010; Muris, Meesters, Aeijkelenboom, & Vincken, 2004) with preserved reliability and validity, yielding a consistent factor structure (Van Roy, Veenstra, & Clench-Aas, 2008), comparable internal consistency and test–retest reliability (Muris, Meesters, & van den Berg, 2003), and demonstrated criterion validity in discriminating between children with and without psychiatric problems (Muris et al., 2004). Because parent and child reports provide valid, though often contrasting, perspectives on internalizing and externalizing psychopathology among children aged between 8 and 16 years (van der Ende, Verhulst, & Tiemeier, 2012), both caregiver and child reports were obtained at baseline and follow-up assessments.

Each SDQ item was rated on a 3-point scale (0 = *not true*, 1 = *somewhat true*, and 2 = *certainly true*), with each subscale constructed as the sum of five items (maximum score = 10). The SDQ scoring algorithm defines scores in the “abnormal” range on each subscale for parent- and for child-reported data (representing approximately the top 10% of the population based on UK normative data), and identifies children with a high probability of meeting clinical thresholds for mental health diagnoses (Goodman, Ford, Simmons, Gatward, & Meltzer, 2003). In community samples, the SDQ psychopathology items load most parsimoniously on internalizing and externalizing psychopathology domains (Goodman et al., 2010; Laurens et al., 2012). Thus, for our study of a longitudinal community sample, the presence of *internalizing psychopathology* was defined as the presence of an abnormal rating (caregiver reported or child reported) on either the emotional symptoms or the peer relationship problems subscales. *Externalizing psychopathology* was defined as an abnormal rating (caregiver reported or child reported) on either the conduct problems or the hyperactivity–inattention subscales. Next, four internalizing and four externalizing trajectories were derived based on ratings obtained from the baseline and follow-up assessments. The four internalizing psychopathology trajectories comprised the following: no internalizing psychopathology (“none”), including children with no internalizing psychopathology at either baseline or follow-up; “remitting” internalizing psychopathology, including children who presented internalizing psychopathology at the baseline assessment, but no internalizing psychopathology at follow-up assessment; “incident” internalizing psychopathology, including children who presented no internalizing psychopathology at baseline, but did so at the follow-up assessment; and “persistent” internalizing psychopathology, including children who presented internalizing psychopathology at both baseline and follow-up assessments. The four externalizing psychopathology trajectories were similarly constructed and consisted of the following: no externalizing psychopathology (none), including children with no externalizing psychopathology at either baseline or follow-up; remitting externalizing psychopathology, including children who presented externalizing psychopathology at the baseline assessment, but no externalizing psychopathology at the follow-up assessment; incident externalizing psychopathology, including children who presented no externalizing psychopathology at baseline, but did so at the follow-up assessment; and persistent externalizing psychopathology, including children who presented externalizing psychopathology at both the baseline and follow-up assessments.

#### PLEs

PLEs were assessed via nine child-report items (Laurens et al., 2007, 2012) that incorporated five items adapted from the Diagnostic Interview Schedule for Children (Costello, Shanahan, Costello, & Angold, 1982; see Table 1). As for the SDQ, each item was rated on a 3-point scale (0 = *not true*, 1 = *somewhat true*, or 2 = *certainly true*). The nine items demonstrate good internal consistency among children aged 9–11 years (Laurens et al., 2007), and all items load on a single latent construct that is distinguishable from internalizing and externalizing psychopathology constructs (Laurens et al., 2012). A comparable, seven-item instrument used to screen paediatric community samples for PLEs has demonstrated good criterion validity between self-reported questionnaire items and clinician-rated psychotic symptoms on diagnostic interview (Kelleher, Harley, Murtagh, & Cannon, 2011). For the present study, a dichotomous variable was created to index PLE presence at follow-up assessment as the outcome variable for logistic regression analyses, defined as a “certainly true” rating on at least one of the nine PLE items. In addition, from the baseline assessment, a continuous measure (total PLE score) was constructed by summing the nine PLE item scores (maximum score = 18); this latter variable was used as a potential confounding factor in the analyses investigating the association between internalizing/externalizing psychopathology trajectories and later PLEs.

**Table 1.** Psychotic-like experience items self-reported by children

1. Some people believe that their thoughts can be read. Have other people ever read your thoughts?
2. Have you ever believed that you were being sent special messages through the television?
3. Have you ever thought that you were being followed or spied upon?
4. Have you ever heard voices that other people could not hear?
5. Have you ever felt that you were under the control of some special power?
6. Have you ever known what another person was thinking even though that person wasn't speaking?
7. Have you ever felt as though your body had been changed in some way that you could not understand?
8. Do you have any special powers that other people don't have?
9. Have you ever seen something or someone that other people could not see?

### Statistical analysis

#### Assessment of follow-up sample retention bias

We determined the effect of sample attrition on the representativeness of the longitudinal (follow-up) sample relative to the community sample that provided data at baseline assessment (constituting 95% of eligible children). That is, child-reported data obtained at the baseline assessment were compared between participants who completed the follow-up assessment and participants completing the baseline assessment only, using seven variables: child age, sex, the presence of an abnormal rating on each of the four self-reported SDQ psychopathology subscales (i.e., emotional symptoms, peer relationship problems, conduct problems, and hyperactivity–inattention), and the presence of at least one certainly true rating among the nine PLE items. Sample selection bias was computed using a Heckman correction (Heckman, 1979). That is, the predicted probability of inclusion in the longitudinal sample (sample selection hazard lamda score) was computed as a function of the variables at baseline assessment that significantly related to completion of the follow-up assessment, using the logistic regression procedure. This hazard score was entered as a covariate factor in adjusted regression models to examine the possible implications of selection effect arising from the pattern of missing data.

#### Association of externalizing and internalizing psychopathology trajectories with later PLEs

A series of logistic regression analyses examined the association between child psychopathology trajectories and the presence of at least one PLE at the follow-up assessment. For the internalizing and externalizing psychopathology trajectories separately, unadjusted regression analyses examined the association of psychopathology trajectory with PLEs at follow-up for the remitting, incident, and persistent trajectories, relative to the trajectory indexing a lack of psychopathology at baseline and follow-up (i.e., none).

Subsequently, the regression analyses were repeated to adjust for the effects of factors potentially related to the exposure (trajectory) or outcome (follow-up PLEs) variables. Selected covariates included child sex, child age at follow-up, time lapsing between baseline and follow-up assessments, and total score on the PLE measure at baseline. Regression models were also extended to test for possible interactive effects between any significant covariates at baseline (e.g., Child Sex × PLEs at Baseline interaction). Only significant covariates (or significant interactions of covariates) were retained in the final adjusted model.

Finally, analyses were repeated to control for any effect of longitudinal sample retention bias on the data, using the Heckman correction (Heckman, 1979). That is, the predicted probability of inclusion in the longitudinal sample (sample selection hazard score) was entered as an additional covariate factor in the adjusted logistic regression analyses.

## Results

### Sample

Follow-up data were provided by 561 children and their primary caregivers. On average, children completed follow-up questionnaires 2.1 years following their baseline assessment (*SD* = 1.4 years, range = 0.5–6.3 years; 95% of the sample provided follow-up data within 4.7 years of initial assessment). The mean age of the longitudinal sample at the follow-up assessment was 12.5 years (*SD* = 1.5 years).

Comparison of the child-reported baseline data from these 561 children relative to the remaining 7,538 sample members who did not participate at follow-up indicated that the follow-up sample significantly overrepresented younger children, longitudinal sample: baseline mean age = 10.3, *SD* = 0.7; remaining children: baseline mean age = 10.4, *SD* = 0.8; *t* (7,777) = 2.0, *p* = .05; underrepresented males, longitudinal sample: 51.0% male; remaining children: 45.9% male; χ^2^ (1, *N* = 8,081) = 10.5, *p* = .001; and underrepresented children who exhibited an abnormal (clinical) level of SDQ conduct problems, baseline conduct problems in longitudinal sample: 13.4%; remaining children: 18.9%, χ^2^ (1, *N* = 8,081) = 10.5, *p* = .001. Thus, the predicted probability of inclusion in the longitudinal sample (sample selection hazard lamda score) was computed as a function of child age, sex, and self-reported conduct problems at baseline assessment using the logistic regression procedure (Heckman, 1979). All analyses are presented both with unadjusted data and following adjustment.

At follow-up, full child reports and caregiver reports were available from 553 children. Among these, 27.5% of children reported ≥1 certainly true response on the nine PLE items (compared to baseline prevalence of 66% in the 8,099 9- to 11-year-old children assessed in the community sample; Downs, Cullen, Barragan, & Laurens, 2013; Laurens et al., 2012); 27.3% presented a child- or caregiver-reported internalizing problem (i.e., an abnormal rating on the emotional symptoms or peer relationship problems SDQ scales; cf. 18.5% at baseline assessment), and 26.4% presented a child- or caregiver-reported externalizing problem (i.e., an abnormal rating on the conduct problems or hyperactivity–inattention SDQ scales; cf. 26.5% at baseline assessment).

The prevalence of internalizing psychopathology trajectories over the approximately 2 years between baseline and follow-up assessments were as follows: none, 60.8%; remitting, 11.9%; incident, 9.9%; and persistent, 17.4%. The prevalence of the externalizing psychopathology trajectories over the corresponding period were as follows: none, 59.5%; remitting, 14.1%; incident, 8.9%; and persistent, 17.5%.

### Association between internalizing trajectories and later PLEs

Table 2 summarizes the unadjusted associations evident between internalizing trajectories (remitting, incident, and persistent; each relative to the no internalizing psychopathology trajectory) and the presence of at least one certainly true PLE at follow-up (including regression coefficients, standard errors, *p* values, odds ratios, and 95% confidence intervals). These analyses revealed statistically significant associations between incident and persistent internalizing trajectories and later PLEs. Children with incident internalizing psychopathology were over three times (odds ratio [*OR*] = 3.4) more likely than children who presented no internalizing psychopathology at baseline and follow-up assessments (none) to exhibit a PLE symptom at the follow-up assessment. Similarly, children presenting the persistent internalizing trajectory were almost three times (*OR* = 2.8) more likely to present a PLE a follow-up than were children in the no internalizing problems trajectory.

**Table 2.** Association of childhood internalizing psychopathology trajectories with PLEs at follow-up in three models

Internalizing Trajectory Comparisons^*b*^	Unadjusted			Adjusted for Significant Covariates			Adjusted for Significant Covariates and Selection Bias^*a*^		
	*B* (*SE*)	*p*	*OR* (95% CI)	*B* (*SE*)	*p*	*OR* (95% CI)	*B* (*SE*)	*p*	*OR* (95% CI)
Remitting vs. none	−0.057 (0.338)	.865	0.944 (0.487–1.832)	−0.647 (0.369)	.079	0.524 (0.254–1.078)	−0.639 (0.368)	.083	0.528 (0.256–1.087)
Incident vs. none	1.235 (0.303)	**<.0001**	**3.440 (1.898–6.234)**	1.082 (0.315)	**.001**	**2.950 (1.591–5.467)**	1.085 (0.315)	**.001**	**2.959 (1.596–5.487)**
Persistent vs. none	1.028 (0.249)	**<.0001**	**2.796 (1.718–4.550)**	0.644 (0.264)	**.015**	**1.905 (1.135–3.196)**	0.641(0.264)	**.015**	**1.898 (1.131–3.183)**

*Note:* PLEs, Psychotic-like experiences; *OR*, odds ratio.

a In all analyses, the contribution of selection bias was nonsignificant. The significant covariate (predicting PLEs at follow-up) that was retained in the adjusted model was child-reported PLEs at baseline assessment, *B* (*SE*) = 0.142 (0.025), *p* < .000001, *OR* (95% CI) = 1.152 (1.097–1.210). Nonsignificant covariates not retained in the final model (all *p*s > .1) included child sex, child age at follow-up assessment, time lapsing between baseline and follow-up assessments, and Sex × PLE interaction.

b Outcome measure is PLE at follow-up.

Adjustment for significant covariates (i.e., total PLEs at baseline), had somewhat different consequences depending on trajectory group status. After adjustment, membership of the incident or persistent internalizing trajectory groups were still associated with a significantly increased risk of PLE at follow-up compared to those in the no internalizing trajectory group. For the remitting trajectory, the adjusted regression coefficient became large and negative, suggesting that in the absence of PLE symptoms at baseline, the remitting trajectory might be associated with a protective effect against later PLEs compared to those in the no internalizing trajectory group. Because this adjusted coefficient for the remitting trajectory was statistically nonsignificant, this apparent protective effect could simply be due to chance. Finally, all the previous associations remained significant after adjustment for selection bias and the significant covariate (Table 2), implying that sample selection bias was unlikely to have influenced study findings.

### Associations between externalizing trajectories and later PLEs

Table 3 reports the unadjusted associations between each externalizing trajectory (remitting, incident, and persistent; each relative to the no externalizing psychopathology trajectory) and the presence of at least one certainly true PLE at follow-up. As with the internalizing psychopathology trajectories, statistically significant associations were identified between the persistent and the incident externalizing trajectories and later PLEs. That is, both children with incident and with persistent trajectories of externalizing psychopathology were over twice as likely (*OR* = 2.3 and 2.6, respectively) as children without externalizing psychopathology at baseline or follow-up to exhibit a PLE at follow-up assessment. The remitting pathway (relative to none) was not significantly associated with PLEs at follow-up.

**Table 3.** Association of childhood externalizing psychopathology trajectories with PLEs at follow-up in three models

Externalizing Trajectory Comparisons^*b*^	Unadjusted			Adjusted for Significant Covariates			Adjusted for Significant Covariates and Selection Bias^*a*^		
	*B* (*SE*)	*p*	*OR* (95% CI)	*B* (*SE*)	*p*	*OR* (95% CI)	*B* (*SE*)	*p*	*OR* (95% CI)
Remitting vs. none	0.421 (0.287)	.142	1.524 (0.868–2.676)	−0.090 (0.318)	.778	0.914 (0.491–1.704)	−0.213 (0.335)	.524	0.808 (0.419–1.558)
Incident vs. none	0.844 (0.323)	**.009**	**2.325 (1.235–4.377)**	0.773 (0.336)	**.021**	**2.166 (1.12–4.182)**	0.761 (0.336)	**.023**	**2.141 (1.108–4.135)**
Persistent vs. none	0.957 (0.249)	**<.0001**	**2.603 (1.598–4.241)**	0.687 (0.280)	**.014**	**1.987 (1.148–3.437)**	0.592 (0.291)	**.042**	**1.807 (1.022–3.194)**

*Note:* PLEs, Psychotic-like experiences; *OR*, odds ratio.

a In all analyses, the contribution of selection bias was nonsignificant. Covariates (predicting PLEs at follow-up) that were retained in the adjusted model included child-reported PLEs at baseline assessment, *B* (*SE*) = 0.132 (0.025), *p* < .000001, *OR* (95% CI) = 1.141 (1.087–1.198), and child sex, *B* (*SE*) = –0.383 (0.212), *p* = .071, *OR* (95% CI) = 0.682 (0.450–1.034). Nonsignificant covariates not retained in the final model (all *p*s > .5) included child age at follow-up assessment, time lapsing between baseline and follow-up assessments, and Sex × PLE interaction.

b Outcome measure is PLE at follow-up.

Adjusted analyses examining the extent to which these significant associations between externalizing trajectories and PLEs at follow-up could be explained by the confounding effects of factors correlated with both externalizing psychopathology and PLEs are summarized in Table 3, along with the covariate factors found to relate significantly with follow-up PLEs that were therefore retained in the adjusted model (i.e., child sex and baseline total PLE score). Analyses revealed no significant effect for the interaction term (Sex × Baseline Total PLE Score), and the interaction term was therefore excluded from the final fitted model. Statistical control for the effects of confounding factors had almost no impact on the strength and direction of effect for both the persistent and the incident pathways in the fully adjusted model.

Finally, comparison of results obtained before and after adjustment for sample selection hazard scores (Table 3) indicated that the conclusions drawn from both sets of findings were similar, thus suggesting that sample selection bias was unlikely to have influenced study findings. In all adjusted analyses (as for unadjusted), no significant association was apparent for the remitting pathway relative to none.

## Discussion

This study sought to determine the association between stability/instability (or continuity/discontinuity) of internalizing and externalizing psychopathology over the transition from childhood to adolescence and the occurrence of PLEs in adolescence. In accordance with our hypotheses, children with persisting internalizing or persisting externalizing psychopathology were twice as likely to report later PLEs relative to those who had never manifest internalizing or externalizing psychopathology, whereas children with remitting internalizing or externalizing pathways were no more likely to report later PLEs than those who had never manifest such psychopathology. Children who developed internalizing psychopathology during follow-up (incident) were over three times, and those developing externalizing psychopathology over two times, more likely to report later PLEs relative to those who had never manifest this psychopathology, respectively. Contrary to our expectations, the strength of prediction for the internalizing/externalizing incident pathways was greater than that observed for the persistent trajectories. All other pathways demonstrated predictive associations for adolescent PLEs in accordance with our study hypotheses.

Our results provide new information about the stability/instability of both internalizing and externalizing psychopathology during the transition from childhood to adolescence, and the impact that these pathways have on PLEs in adolescence. Our findings might be interpreted within the context of a cognitive model for the de novo development of PLEs/psychotic symptoms postulated by Garety, Kuopers, Fowler, Freeman, and Bebbington (2001), and developed further by Kuipers et al. (2006). The model outlines a process whereby a varied biopsychosocial predisposition (i.e., comprising multiple genetic and nongenetic factors) renders an individual vulnerable to the effect of a stress-inducing trigger event. The model emphasizes the postulated role that negative emotional processes (arising as a result of a stressful trigger and/or a cognitive dysfunction or impaired information processing) have on generating and then amplifying misappraisals of experiences. If the misappraisal is not discarded as insignificant or is deemed personally significant, or if the appraisal cannot be weighed against appropriate normalizations and is congruent with the schemas held by the individual concerning himself and the people and world around him, there is more chance that the misappraisal will progress to a psychotic symptom. This model has been used to structure investigation into factors affecting PLE severity in clinical samples of children as young as 8 years old. For example, Ames et al. (2014) reported significant correlations among the severity of PLEs and cognitive bias, emotional disturbance, and negative life events in children aged 8–14 years. While our findings do not, in themselves, allow direct inference regarding the mechanisms underlying the observed predictions, the cognitive model suggests possible avenues for future investigation of likely mechanisms. In our study, there was a more than twofold increased risk of PLEs in those who had persistent internalizing psychopathology. It is possible, though unlikely, that such psychopathology occurs prior to PLEs purely as a result of internalizing and PLEs sharing components of the aforementioned biopsychosocial vulnerability but arising at different points in development. Alternatively, internalizing symptomology likely acts to increase the likelihood of misappraisal (Garety et al., 2001), because internalizing disorders are themselves characterized by negative schemas of self, others, and the world, which directly impact the process of appraising stimuli and have been found to predispose adults to develop psychosis in the context of depression (Krabbendam et al., 2005) and increase the severity of psychotic symptoms in acutely relapsed adults (alongside depressed mood and poor self-esteem; Smith et al., 2006). It is of note that poor mental health, as measured by the SDQ in young people across geographically diverse regions, is associated with increased risk of bullying (Analitis et al., 2009). Bullying in turn is a likely predisposing factor to PLEs (Campbell & Morrison, 2007; Mackie et al., 2013), warranting further study of the complex interplay of vulnerability, stressors, and psychopathology, particularly around the transition from childhood to adolescence. The even greater (threefold) increase in risk for PLEs in those developing internalizing psychopathology during the follow-up period potentially is a later-developing variant of the same process, thus presenting more acutely at follow-up. Alternatively, these children might represent a more severely affected subset whose emotional problems are happening as part of the development of an evolving psychotic disorder rather than providing a medium facilitating the development of psychotic symptoms. The review of studies reported by Kelleher et al. (2012) noted that the incidence of PLEs in adolescence decreased but were more likely to be associated with nonpsychotic comorbid psychopathology with increasing age.

Externalizing psychopathology has received relatively less research attention than internalizing problems with respect to pathogenesis of psychotic symptoms, though the magnitude of prediction observed in this study is similar. The cognitive model provides a helpful framework to explore causal mechanisms. It may be that what we have reported represents two separate processes as a result of a single risk factor. The retrospective finding (Peralta et al., 2011) that 17% of adults suffering from a first-episode schizophrenia spectrum disorder had been diagnosed with attention-deficit/hyperactivity disorder in childhood was attributed to both disorders being manifestations of obstetric complications and neurodevelopmental delay. Though that study relates to first-episode psychotic illness, it is possible that comparable factors are in operation in relation to PLEs. As previously noted, externalizing psychopathology is characterized by “disinhibition,” affecting attentional processing and impulse control, which may make it harder for a child to appraise external stimuli appropriately and hold in mind alternative explanations for their occurrence, thereby amplifying the cognitive dysfunction. Not only do overlapping risk factors likely affect a child's predisposition to externalizing psychopathology and PLEs, but a combination of these factors may act to make progression of a misappraisal increasingly likely and maintain PLEs once formed. In a comprehensive review of risk factors involved in externalizing disorders, parental stress, stressful life events, social isolation, harsh discipline, and physical abuse were identified as influential factors for externalizing disorders that overlap as risk factors for PLEs (Deater-Deckard, Dodge, Bates, & Pettit, 1998). In particular, as a result of harsh parenting, further cited as a predictor of externalizing symptoms (conduct problems; Bayer et al., 2011), it is possible that a child would be at greater risk of perceiving themselves as vulnerable (and, conversely, others as dangerous) and having negative schema around themselves and the world, which, while manifesting in angry, inattentive, or impulsive externalizing-type responses to life situations, would predispose the child to the development of PLEs as per Garety et al.'s model (2001). Antisocial behavior occurring as a result of an externalizing psychopathology might increase the risk of social isolation (excepting contact with similarly socially deviant peers with comparable cognitive distortions), thus maintaining misappraisals and further increasing the likelihood of PLEs occurring.

The incident group of children with externalizing psychopathology may represent a later adolescent-onset occurrence of conduct problems (Moffitt & Caspi, 2001; Moffitt, Caspi, Dickson, Silva, & Stanton, 1996) or perhaps a milder form of hyperactivity–inattention manifesting as academic demands intensify. Though the baseline (childhood) externalizing psychopathology was absent or subthreshold, the twofold increased risk of PLEs seen in this group may nonetheless have evolved in a similar manner to the persisting trajectory group. It is also possible in this London community sample that illicit drug use had begun during the follow-up period, particularly in the groups with persisting or incident externalizing psychopathology, exerting its known effect on PLEs (Mackie et al., 2013).

Those individuals whose childhood internalizing or externalizing psychopathology remitted within the follow-up period did not, according to our stated hypothesis, demonstrate increased risk for adolescent PLEs. It is possible that the remitting group of youth reported internalizing or externalizing psychopathology at baseline that was less severe than those individuals in the persisting group, and therefore more likely to attenuate during the follow-up period. Alternatively, it is possible that some of our sample were more able to reflect and reclassify normative experience at follow-up. However, in light of prospective samples demonstrating cross-sectional but not predictive relationships between depressive symptoms and PLEs in teenagers and young adults (Wigman et al., 2011), our remitting findings suggest similar possibilities in younger age ranges. It is a reminder that our findings are at best inferential in a context of symptomatic heterogeneity and developmental change.

Our discussion of how internalizing and externalizing symptoms might contribute to the development of PLEs in the context of a cognitive model (Garety et al., 2001; Kuipers et al., 2006) does not intend to negate the evidence for a diverse range of genetic (Schizophrenia Working Group of the Psychiatric Genomics, 2014) and nongenetic factors (Matheson, Shepherd, Laurens, & Carr, 2011) that contribute to the aetiology of schizophrenia.

Overall, the prevalence rates for any form of internalizing or externalizing psychopathology (whether remitting, incident, or persisting) in our community sample are higher than rates of diagnoses that were cited in our brief summary of the epidemiology of psychopathology in young people across geographically distinct world regions. Our use of screening measurements, including self-rating, might have permitted deeper insight into levels of morbidity. Another possibility is that our application of the normative cutoff for the threshold defining disorder level for psychopathology on the SDQ screening tool (~10%; Goodman et al., 2003) within this community sample may have been too generous, thus allocating children to incident, remitting, and persistent patterns inappropriately, and artificially inflating these subgroup sizes. Alternatively, these rates may in part reflect the cohorts' origins from deprived inner-city communities in Greater London.

While our findings indicate that persistence and incidence of internalizing and externalizing psychopathology in late childhood and early adolescence are associated with increased likelihood of later PLEs, these trajectories in isolation offer limited prospects for preventive intervention for psychoses specifically. Internalizing and externalizing disorders are antecedents of diverse adult psychopathology outcomes, not solely psychotic illness (Copeland et al., 2009; Kim-Cohen et al., 2003). We have previously proposed a method of using internalizing and externalizing psychopathology in combination with other replicated antecedents of schizophrenia to delineate children who may be more specifically at risk for psychotic disorders (Laurens, Hodgins, Taylor, & Murray, 2011). It is likely that these children will require tailored interventions addressing these co-occurring difficulties (e.g., Maddox et al., 2013). Further research is needed into the factors that influence the continuity of internalizing and externalizing psychopathology that co-occurs with PLEs and other antecedents of schizophrenia.

The study had several limitations. PLEs were deemed present if the young person rated one of nine items as certainly true. Though all nine items load on a single latent construct, it is known that the items do not load with equal strength or assess the latent construct equivalently (Laurens et al., 2012). The baseline measurement phase was unavoidably protracted, which led to variability in duration of follow-up (for which adjustments were incorporated in the analysis). It is also possible that pubertal status exerted a variable confounding effect, though assessment of, and adjustment for, this factor was impractical in this epidemiological investigation.

The strengths of this study were multiple. We elected to sample a community cohort prospectively during the transition from childhood to adolescence, thereby avoiding biased recollections of historical behavior. Validated questionnaires, completed by multiple informants (self and caregiver) captured both the internal experiences of a young person (necessary for investigation of internalizing psychopathology and PLEs) and external observation of behavior (advocated in assessing externalizing symptoms). We adjusted for potential confounders such as age and sex of the child, and the presence of PLEs at the baseline assessment. The examination of relationship between externalizing psychopathology and PLEs was a key strength in view of the paucity of studies investigating this association relative to internalizing psychopathology and PLEs (Downs et al., 2013).

Our findings demonstrate that the persistence or incidence of both internalizing and externalizing psychopathology in late childhood and early adolescence is associated with the later occurrence of PLEs. Future research might valuably employ a cross-sequential design incorporating several developmental transition periods (e.g., childhood to early adolescence, early to late adolescence, and late adolescence to young adulthood) to determine whether the greater associations observed for the incident psychopathology trajectories with PLEs reflect a specific age-period effect or may be due to a tendency for psychopathology to co-occur in time. The comparable strength of prediction arising from both the persistence and de novo emergence of externalizing problems raises awareness of the importance of not limiting our focus to internalizing psychopathology as a causative factor in the development of PLEs and psychotic illness. Furthermore, the results encourage investigation into the processes causing and contributing to disadvantageous trajectories of psychopathology in children and adolescents as they develop, in the hope that their impact on their future mental health might be mitigated. Further research is needed to elucidate the most effective means of altering these adverse trajectories.
